# Real‐World Data and Real‐World Evidence in Regulatory Decision Making: Report Summary From the Council for International Organizations of Medical Sciences (CIOMS) Working Group XIII

**DOI:** 10.1002/pds.70117

**Published:** 2025-03-11

**Authors:** Sean Hennessy, Yoshiko Atsuta, Sanna Hill, Lembit Rägo, Juhaeri Juhaeri, Council for International Organizations of Medical Sciences (CIOMS) Working Group XIII, Alteri Enrica, Atsuta Yoshiko, Aubrun Elodie, Azoulay Laurent, Baumfeld Andre Elodie, Blackburn Stella, Boerstoel Mariette, Brookland Thomas, Campbell Ulka, Crane Gracy, Goettsch Wim, Gomez‐Caminero Andres, Gomez‐Reino Elisa, Haenisch Britta, Hennessy Sean, Heß Steffen, Hill Sanna, Irs Alar, Ishiguro Akihiro, Iyasu Solomon, Jonsson Funk Michele, Juhaeri Juhaeri, Junji Moriya, Lambert Laurie, Li Jie, De Luise Cynthia, Machlitt Andrea, Mayer Miguel‐Angel, Mera Robertino, Nishioka Kinue, Nomura Manami, Nonaka Takahiro, Rägo Lembit, Rubino Heather, Sato Daisaku, Schiel Anja, Shaw David, Soares Monica, Stingl Julia, Townend David, Wakao Rika, Wang Shirley, Wicherski Julia, Wormser David, Zint Kristina

**Affiliations:** ^1^ Center for Real‐World Effectiveness and Safety of Therapeutics (CREST) University of Pennsylvania Perelman School of Medicine Philadelphia Pennsylvania USA; ^2^ Department of Biostatistics, Epidemiology and Informatics University of Pennsylvania Perelman School of Medicine Philadelphia Pennsylvania USA; ^3^ Japanese Data Center for Hematopoietic Cell Transplantation Nagoya Japan; ^4^ Aichi Medical University School of Medicine Nagakute Japan; ^5^ Council for International Organizations of Medical Sciences (CIOMS) Geneva Switzerland; ^6^ Sanofi Bridgewater New Jersey USA

**Keywords:** decision making, lifecycle, medicine, public health, real‐world data, real‐world evidence, regulation

## Abstract

Data from sources other than traditional randomized clinical trials are known as real‐world data (RWD), and the evidence derived from the review and analysis of RWD is known as real‐world evidence (RWE). RWD and RWE are used increasingly throughout the lifecycle of medicinal products to provide evidence about their effectiveness and safety. Recent regulatory guidance about RWE and approvals based on the use of RWE to demonstrate beneficial effects of products have created an urgency to develop generally accepted processes that promote trust in the evidence‐generation process. A recent report from a working group of the Council for International Organizations of Medical Science (CIOMS) describes the use of RWE for decision making in the lifecycle of medical products, describes RWD and data sources, discusses key scientific considerations in the generation of RWE, and discusses ethical and governance issues related to the use of RWD. This paper provides a high‐level summary of this report. More work remains to be done to globally harmonize practices and guidance for using RWD and RWE for regulatory decision making, thereby maximizing the benefits they can bring to patient care and public health.

**Summary**

- In recent years, real‐world evidence use has evolved, driven by recognition and acceptance by regulators, payers, and health technology assessment bodies to answer specific research questions.
- Each real‐world data source has its strengths and limitations, and scientific evaluation of the fitness of a real‐world data source for a given study is essential in choosing a data source.
- Study design decisions affect the validity and generalisability of the study results and thus are essential to the generation of fit‐for‐purpose real‐world evidence.
- There is an urgent need for regulators to provide principles and for regulators to come together to harmonize the approach taken on ethics and governance issues.
- More work remains to be done to globally harmonize practices and guidance for using real‐world data and real‐world evidence for regulatory decision making.

## 1 Introduction

Randomized controlled trials (RCTs) are considered the gold standard design for evaluating the benefits of medicinal products. Randomization, the key feature of RCTs, provides a mechanism by which randomized groups are balanced, on average, with respect to baseline factors, both measured and unmeasured. Beginning with the enactment of the 1962 Kefauver–Harris Drug Amendments to the US Food Drug and Cosmetic Act and analogous laws in other countries, RCTs became the norm for demonstrating efficacy [1]. However, a number of important research questions are challenging to address with randomized designs, such as the study of rare adverse effects, long‐term drug effects, and the treatment of rare diseases and other conditions where patients may be reluctant to be randomized. Further, many pre‐approval trials have enrolled participants who were not fully representative of the population who used the product or were conducted under conditions that do not represent those under which the new product would be used in typical health care settings once it was approved. This tendency led to concerns about an *efficacy‐effectiveness gap* between outcomes observed in RCTs (efficacy) compared with real‐world circumstances (effectiveness) [2]. Such concerns have led to increasing use of real‐world data (RWD) and real‐world evidence (RWE), defined below, to inform regulatory and clinical decisions about medical products.

While various definitions of RWD have been proposed (see Table 1, for examples), there is currently no consensus definition. However, a recent International Council for Harmonization of Technical Requirements for Pharmaceuticals for Human Use (ICH) Reflection Paper [9] indicates a willingness on behalf of ICH to start creating harmonized guidance, including terms and definitions for this area.

**TABLE 1:** Some definitions of real‐world data.

Organization	Definition of RWD
US Food and Drug Administration [3]	Data relating to patient health status and/or the delivery of health care routinely collected from a variety of sources. Examples of RWD include data derived from electronic health records (EHRs), medical claims data, data from product or disease registries, and data gathered from other sources (such as digital health technologies) that can inform on health status.
European Medicines Agency [4]	Routinely collected data relating to a patient's health status or the delivery of health care from a variety of sources other than traditional clinical trials.
Joint International Society for Pharmacoepidemiology (ISPE) – International Society for Pharmacoeconomics and Outcomes Research (ISPOR) Special Task Force on Real‐World Evidence in Health Care Decision Making [5]	Data obtained outside the context of randomized controlled trials (RCTs) generated during routine clinical practice.
International Society for Pharmacoeconomics and Outcomes Research (ISPOR) [6]	Data relating to areas such as patient health status and/or healthcare delivery not collected in conventional RCTs. Examples of RWD are EHRs; wearables; medical claims data; surveys; and product, patient, and disease registries.
RAND Corporation [7]	Data collected during the routine delivery of care and its reimbursement. This type of data, referred to as real‐world data, includes patient registries, EHRs, healthcare claims databases, and patient‐generated data and is defined by its production outside of a research study.
Innovative Medicines Initiative Get Real Project [8]	An umbrella term for data regarding the effects of health interventions (e.g., safety, effectiveness, resource use) that are not collected in the context of highly‐controlled RCT's. Instead, RWD can either be primary research data collected in a manner which reflects how interventions would be used in routine clinical practice or secondary research data derived from routinely collected data. Data collected include, but are not limited to, clinical and economic outcomes, patient‐reported outcomes (PRO) and health‐related quality of life (HRQoL). RWD can be obtained from many sources including patient registries, electronic medical records, and claims databases.

RWE is evidence derived from the review and/or analysis of RWD [10].

The Council for International Organizations of Medical Sciences (CIOMS) recently issued a consensus report [11] to inform discussions about the use of RWD and RWE for regulatory and health care decision making, product authorization, reimbursement, and clinical use. This review provides a high‐level summary of that report.

### 1.1 Real‐World Evidence for Decision Making During the Product Lifecycle

**FIGURE 1:**
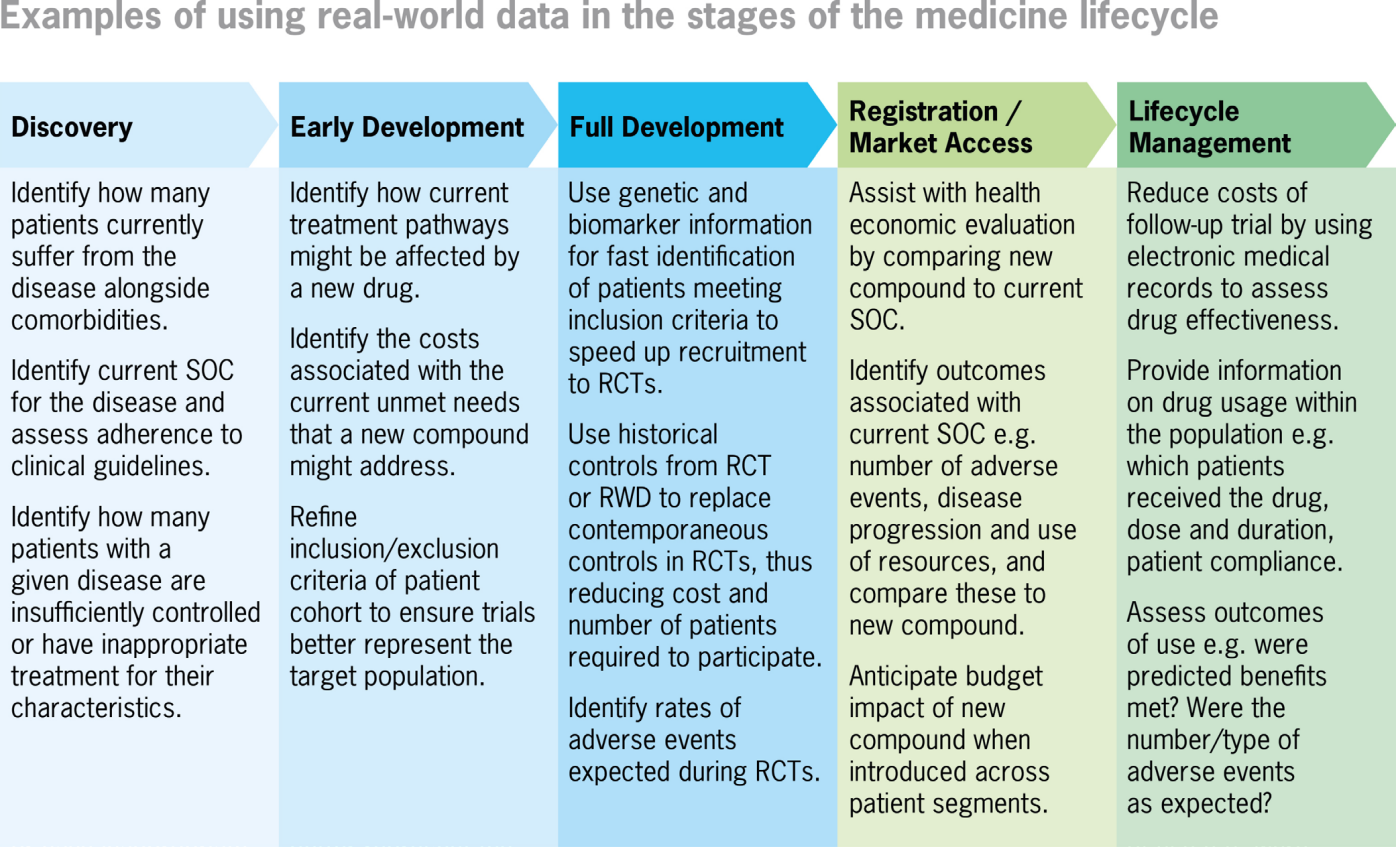
Examples of using real‐world data in the stages of the medicine lifecycle. *Note:* This figure has been adapted from the original figure in the CIOMS WG XIII report. RCT, randomized controlled trial; SOC, system organ class. *Source:* Orpha Strategy.

RWE can be used to inform a broad range of regulatory, coverage, and clinical decisions by various kinds of stakeholders, including patients, clinicians, regulators, health technology assessment (HTA) agencies, healthcare payers, and biopharmaceutical and medical device companies. RWE has a key role to play in supporting decision‐making along the lifecycle of medicinal products, as illustrated in Figure 1 [12, 13].

For RWE to support regulatory decision making, relevant stakeholders should implement a transparent process of planning, reporting, and assessing RWE. A structured template for planning and reporting on the implementation of RWE studies (STaRT‐RWE) has developed a such a template for RWE studies of the safety and effectiveness of treatments [14]. A proposal for HARmonized Protocol Template to Enhance Reproducibility (HARPER) can facilitate study protocol development and enhance transparency and reporting [15].

## 2 Sources of Real‐World Data

RWD from existing health care databases, including insurance claims, electronic health records (EHRs), and registry databases is widely accepted and has been used for decades, mainly for safety evaluation, risk management, and to support benefit–risk evaluation of medicinal products [16, 17]. These RWD sources have many strengths, including longitudinality, large population size, and ready accessibility of the data, allowing for faster study completion. Their limitations include the unavailability of information on key confounding factors and that populations are often limited to patients with certain conditions or exposed to specific medicinal products.

Those considering any given RWD source can evaluate if the RWD study population would contribute clinically meaningful information to guide their decision‐making for some populations of interest, even if not all subgroups of interest. When existing data sources are not suitable to answer the questions at hand, either because of a lack of information or under‐representativeness of the study population, new RWD with primary data collection, or with data collected on an ad‐hoc basis specifically for the study, may be needed. Because the data are collected specifically to answer a set of study questions, these data sources can potentially be more effective in answering those questions.

The availability of many different RWD sources presents a unique opportunity to perform the same study using different sources. A set of organized but distinct RWD sources analyzed separately using the same protocol for the same study is usually called a federated system [18, 19, 20, 21, 22, 23, 24, 25]. The use of multiple databases can enlarge the sample size, broaden representativeness, enrich the data, and prolong the study follow‐up time.

In addition to existing health care data and data collected on research participants recruited by clinicians, direct enrolment of participants by health systems and others without clinician involvement is becoming more common. For example, the ADAPTABLE (Aspirin Dosing: A Patient‐Centric Trial Assessing Benefits and Long‐Term Effectiveness) trial recruited participants directly through electronic health records and patient portals and randomized them to either 81 or 325 mg of daily aspirin, independently of health care providers. All study visits were completed within the web portal and did not require clinic visits [26].

Other important RWD sources are spontaneous reporting systems (SRSs) that record individual case reports that are spontaneously reported or solicited. One of the limitations of SRSs is that all observations reflect reported events, whereas the populations from which these events arise are not known. Other weaknesses include under‐reporting, stimulated reporting, differential reporting, and poor data quality in terms of validity and completeness [27, 28]. While SRSs are usually not an ideal basis for causal inference, they have been an important source for signal detection since the 1960s [29, 30] and are likely to remain so for the foreseeable future.

Cross‐sectional survey databases, such as the US National Health and Nutrition Examination Survey database, are other RWD sources that can play a key role in the evaluation of the burden or prevalence of diseases. Because survey participants are often systematically sampled to be representative of the population, estimates of prevalence can be generalized to that population.

The introduction of new technologies, such as those related to remote care, and the increased use of mobile devices, have provided new sources of information that can be generated with unprecedented volume, speed, and complexity and require a different set of data management and analytical methods. These emerging RWD sources include biosensor data, patient experience data, curated EHRs combined with ancillary data, text or images from radiology information systems, output from laboratory information systems, and output from structured genomics investigation. Although the utility of these emerging sources is still becoming apparent, with the rapid development of modern computing and advanced analytics, it is just a matter of time before they will also be used as key RWD sources in the context of regulatory decision making.

## 3 Real‐World Evidence for Regulatory Use: Key Considerations

In each phase of the product lifecycle, research questions for the inclusion of RWE may arise. The research question of interest is defined considering the evidence gaps, followed by the selection of an appropriate data source (primary or secondary), a study design, and a protocol [31].

### 3.1 Assessing Real‐World Data to Generate Evidence

Scientific fitness‐for‐purpose is critical for the selection of one or more databases. The attributes of a RWD source need to be suitable for the research question or study purpose, including the size and representativeness of the study population and the availability of key variables about exposures, outcomes, and covariates, including confounders with the method of data collection. A single database may not be sufficient for a given research question, and multiple databases may be needed. If so, the principles of scientific considerations of fitness‐for‐purpose apply to each database [32]. When multiple data sources are used, proper work processes for data ingestion and harmonization of datasets into a common data model are extremely important. If data linkage is needed, the right data elements for patient linking must be present. A data feasibility assessment framework called the Structured Process to Identify Fit‐For‐Purpose Data is a systematic process for assessing feasibility to determine if a data source is fit for decision making [30].

The US Food and Drug Administration's (FDA's) draft guidance requests sponsors that submit non‐interventional studies for regulatory review to take responsibility for all activities related to the design, conduct, and oversight of the studies [33]. Data quality frameworks provide structured approaches to assess the quality of RWD [32, 33, 34]. The recent European Medicines Agency framework emphasizes several key dimensions of data quality that are crucial for evaluating the reliability and usability of RWD, including reliability, extensiveness, coherence, timeliness, and relevance [34].

### 3.2 Study Design and Methods

The choice of study design depends on the research question, type, and availability of relevant data, and feasibility of the study. The strengths and limitations of different study designs must be carefully considered to ensure the validity of the study results. RWD/RWE can address research questions like estimating disease incidence/prevalence for orphan medicines, measuring background adverse event rates, selecting comparators for single‐arm studies, and providing descriptive evidence to support label changes. RWD can also be used to explore treatment patterns, healthcare resource use, and patient‐reported outcomes, enriching regulatory decision making beyond safety and efficacy assessments. Valid design choices are essential to ensure the validity of a study's findings, and errors in design are unlikely to be remediable in a study's analysis. Emulating a hypothetical randomized trial for designing studies using RWD is an approach that seeks to address the limitations of nonrandomized studies in evaluating the safety and effectiveness of medical interventions. There are several advantages to this approach, including that it clarifies thinking while making crucial design decisions such as inclusion criteria, duration of follow‐up, and study endpoints, and reduces the potential for introducing error [35]. Given its advantages, target trial emulation is becoming more common and accepted, although it is not always straightforward to conceptualize every research question as an emulated trial.

The successful implementation of a real‐world study hinges on identifying the population that would most benefit from a given therapy or intervention. Identifying a clinically relevant anchor point in time is critical, as it establishes the temporality between potential confounders, the exposure, and the outcome. Historical controls differ from contemporaneous controls in terms of timing for cohort entry. A contemporaneous control arm can be created if all of the RWD was generated on or after the first patient receiving the study agent was enrolled. To account for any potential temporal changes in disease treatment or outcomes, contemporaneous control cohorts are preferable to historical controls. Constructs such as race and ethnicity merit additional care in the design and analysis of studies to generate RWE. The use of race or ethnicity and decisions regarding the representation of those who provide these data should be informed by an understanding of the community's interest in seeing themselves in the results, while respecting privacy concerns. As best practices are evolving in this area [36], researchers are advised to seek up‐to‐date expert guidance on measurement, analysis, and reporting of race or ethnicity [37].

Outcome definitions of RWD studies refer to the specific endpoints or measures that are used to evaluate the effectiveness or safety of a particular intervention or exposure in the study population. Selecting a clinical outcome measure in the real‐world assessment of medicine effectiveness and safety involves careful consideration of disease or condition factors and data sources [38]. Outcome definitions described in the CIOMS report include clinical, patient‐reported, surrogate, and economic outcomes. Composite endpoints, with two or more component endpoints, are often used as study endpoints when the individual events included in the score are rare, and/or when it makes biological and clinical sense to group them. Using composite endpoints in RWD studies confers advantages by amalgamating multiple, related outcomes. Selecting the appropriate exposure definition is critical in the real‐world assessment of medicine effectiveness and safety. The “as‐started” exposure definition, which is analogous to the intention‐to‐treat principle in RCTs, follows patients from the start of their treatment until the end of follow‐up, regardless of treatment discontinuation [39]. This exposure definition answers the clinical question of whether to initiate a medicine versus another; it is about the intent of treatment initiation. In the time‐varying exposure definition, patients are followed from a cohort entry point, and their exposure status is allowed to vary over time.

Confounding is one of the biggest challenges in working with RWD and plays an even more significant role when comparing treatment effectiveness with safety. A confounder can be defined as a variable for which control by design or analytical adjustment is required to obtain unbiased estimates of the effect of an exposure on the outcome under study. In nonrandomized studies and hybrid clinical trial designs, it is important to assess the potential impact of residual confounding. When using RWD, assessment of systematic error (bias) is critical for a study aimed at evaluating a medicinal product's treatment effect. The role of bias in epidemiology and pharmacoepidemiology has been described in many guidelines and reference works.

Pragmatic randomized clinical trials are largely thought to answer important and relevant questions about the real‐world effects of treatments in post‐approval routine clinical practice settings [40]. They typically include a broader and more diverse study population of patients who are eligible to receive study interventions as part of routine clinical practice. Research participants are recruited from clinical practice settings. Primary and secondary outcomes could be collected from claims or EHRs, or collected through limited electronic case report forms, with or without adjudication. While such trials can incorporate pragmatic elements, they can still have features to maintain rigorous standards for data collection.

External controls, often derived from past traditional trials, have been used as a control arm for single‐arm trials. More recently, external controls derived from RWD are increasingly being used as controls for single‐arm trials, especially for serious and rare diseases where a trial is not feasible or where randomization is unethical [41]. Regulations and guidance documents have indicated circumstances where historical control arm designs can be used. US Code of Federal Regulations 21CFR 314.126 [42] indicates that historical control designs are usually reserved for special circumstances. ICH E10 (2001) [43] describes selection strategies for control groups in clinical trials intended to demonstrate efficacy. The US FDA has issued a guidance document on externally controlled trials [41, 44].

### 3.3 Real‐World Evidence Reporting‐Transparency and Trust

Recent regulatory approvals based on RWE created an urgency to develop generally accepted processes that promote trust in the evidence‐generation process [45]. Registration of RWD studies has been proposed to improve transparency, trust, and research replicability. In the CIOMS report, quality tools for RWD studies and RWE reporting are introduced [46, 47, 48, 49].

In 2020, an extensive US‐based study team launched the RCT DUPLICATE initiative (Randomized, Controlled Trials Duplicated Using Prospective Longitudinal Insurance Claims: Applying Techniques of Epidemiology) to compare the findings of RCTs relevant to regulatory decision making with the findings of noninterventional RWE that emulate the trial design as closely as possible in a consistent, transparent, and reproducible process that would be acceptable to regulators [50, 51]. Some of the main takeaways from RCT DUPLICATE included: (1) simple measures of agreement in results between RCTs and RWD studies lack nuance and will not tell the whole story; (2) while residual bias or random error is always a potential explanation for observed divergence in results between a trial and a RWD study, when the divergence is driven by design emulation differences, the database study could be accurately targeting a different effect (for a different research question) than the trial; (3) given low adherence in clinical practice, it can be challenging to replicate trial findings for outcomes with a long induction window or time varying hazard over extended follow up; and (4) while comparisons of RCT and RWD studies typically use the result of a single trial as a reference standard, this does not take into account the uncertain replicability of a trial's findings even by other trials. Other similar RCT emulation projects have also found that when the data and design are fit‐for‐purpose, non‐randomized database studies can reach similar conclusions about drug effects as randomized trials [47, 48, 49, 50, 51, 52]. The real benefit of non‐randomized, non‐interventional RWD studies is in how they can complement rather than replace evidence from RCTs in extending knowledge about the effects of medical products in post‐approval settings.

## 4 Ethics and Governance

An important ethical issue is the efficacy‐effectiveness gap, the difference between the efficacy observed in clinical trials and the effectiveness observed in real‐life settings. The broader use of RWE to evaluate efficacy as well as safety is justified not only by a need for stronger and broader evidence and to include neglected groups in the evidence base [53], but also by concerns that evidence from RCTs sometimes does not translate well into real‐world use. Trials also tend to under‐report harm, given the narrow eligibility that limits exposure to certain groups until safety is proven [54]. Evidence shows that the efficacy‐effectiveness gap worsens disease response and survival outcomes and increases toxicity in the clinical setting [55]. This gap provides an ethical argument to incorporate RWE into decision making.

Other ethical and governance issues discussed in the report are informed consent, privacy, and data protection, as summarized below.

### 4.1 Consent

Perhaps the most important ethical issue concerning the use of RWD is informed consent. In many cases, patient data is routinely used for service evaluation and audit without explicit consent being sought. For example, some healthcare providers in the United Kingdom simply display posters informing patients about this. If RWD is to be used more, then routine data linkage with patient records for research, in addition to evaluation and audit, may be a next step, which raises some issues regarding confidentiality and privacy. However, given that such data are often used without explicit consent for other purposes, it might be argued that seeking active consent for research use is disproportionate given the potential benefits of research. The need for consent for clinical trials comparing standard‐of‐care interventions has sparked significant debate. While some argue that all trials should require comprehensive consent, others contend that trials limited to standard treatments do not need consent, since the interventions are already accepted practices [56].

### 4.2 Privacy and Data Protection

The purpose of data protection legislation is to protect the fundamental rights and interests of citizens in relation to the processing of personal data that relates to them. On the one hand, this can be satisfied in many situations where sensitive personal data about individuals are processed, for example, in relation to banking details or in other commercial transactions that place citizens in vulnerable situations in relation to their personal data, through the safeguard of a clickwrap or click‐to‐sign consent. On the other hand, highly regulated areas such as medical research, with multiple safeguards and independent scrutiny, are made almost impossible to negotiate. RWD is in danger of being so restricted by data protection laws and regulations that it becomes impossible to work with, whereas in practice it is an area where the interests of individual citizens are robustly protected, more so than in many commercial situations imposed on consumers, and where the outcomes that the RWD research pursues are clearly in the public interest and in the interests of protecting human dignity.

### 4.3 Broad Data Protection Landscape

From its common international roots in the late 1970s [46], data protection law has shared a common language and basic shape [57]. The underpinning idea is that the individual citizen has human rights, particularly privacy rights in relation to the processing of their personal data. According to the European Union's General Data Protection Regulation (EU GDPR 2016/679), lawful processing is prescribed to include, although not exclusively, two fundamental elements:

1. Processing must be on the legal bases for the processing of personal data; and
2. Data subjects must be given information about the identity and contact details of the data controller and the purpose and nature of the processing of the personal data.

Data protection and processing are the major unresolved conceptual and technical issues for the use of RWD. Strong data protection law should not be seen as an obstacle or barrier to the effective processing of personal data, and therefore, where there are unresolved technical issues, they must be resolved. A well‐functioning personal data protection regime is essential to the acceptance and operation of RWD processing.

### 4.4 Information Provision

Separately from the requirement for a legal basis for personal data processing, those who process personal data must inform the data subjects of their identity, contact details, and the purpose for and nature of the processing they propose. This is not a requirement for informed consent in all cases. This requirement acknowledges that the data subject has rights that they can only engage when they are aware that processing is taking place. It allows, in certain circumstances, for data subjects to opt out or modify their participation in certain processing and is therefore a necessary part of the process.

### 4.5 De‐Identifying the Data

Data protection law operates only on personal data, meaning data that identify an individual natural person or that are capable of doing so when linked to other data. The easiest example to comprehend is pseudonymised data. Personal data have certain identifiers, for example, a name or address, which are replaced with a code. “Pseudonymization” of data means replacing any information that could be used to identify an individual with a pseudonym or, in other words, a value that does not allow the individual to be directly identified. The level of pseudonymising needed to reasonably protect persons from being identified without hindering researchers' ability to address questions of clinical and public importance merits further consideration.

There is an urgent need for regulators to provide principles, and for governments to come together to harmonize the approach taken on ethics and governance issues. The lack of guidance at least gives an opportunity for strong guidance to be created now to fill the gaps.

## 5 Conclusions and Future Directions

In recent years, RWE has evolved, driven by recognition and acceptance by regulators, payers, and HTA bodies to answer specific research questions.

Each RWD source has its strengths and limitations, and scientific evaluation of the fitness of a RWD source for a given study is essential in choosing a data source. Study design decisions affect the validity and generalisability of the study results, and thus are essential to the generation of fit‐for‐purpose RWE. Ethics and governance issues concerning RWD and RWE include the efficacy‐effectiveness gap, inferred consent, and privacy and data protection. The ethical frameworks and legislation must be established to enable RWE to be used in regulatory decision‐making.

More work remains to be done to globally harmonize practices and guidance for using RWD and RWE for regulatory decision making.

Members of the Council for International Organizations of Medical Sciences (CIOMS) Working Group XIII.

**Table:** 

Name	Company/Organization	Country
Alteri, Enrica	Formerly European Medicines Agency (EMA)	Switzerland
Atsuta, Yoshiko	Japanese Data Center for Hematopoietic Cell Transplantation/Aichi Medical University School of Medicine	Japan
Aubrun, Elodie	Novartis Pharma AG	Switzerland
Azoulay, Laurent	McGill University	USA
Baumfeld Andre, Elodie	Roche	USA
Blackburn, Stella	IQVIA	UK
Boerstoel, Mariette	Bristol Myers Squibb	USA
Brookland, Thomas	Roche	USA
Campbell, Ulka	Pfizer	USA
Crane, Gracy	Roche	UK
Goettsch, Wim	National Health Care Institute, Diemen/Utrecht University	Netherlands
Gomez‐Caminero, Andres	Merck Sharp & Dohme	USA
Gomez‐Reino, Elisa	Alexion	USA
Haenisch, Britta	Federal Institute for Drugs and Medical Devices (BfArM)	Germany
Hennessy, Sean	University of Pennsylvania	USA
Heß, Steffen	Federal Institute for Drugs and Medical Devices (BfArM)	Germany
Hill, Sanna	Council for International Organizations of Medical Sciences (CIOMS) Switzerland	Switzerland
Irs, Alar	State Agency of Medicines	Estonia
Ishiguro, Akihiro	Pharmaceuticals and Medical Devices Agency (PMDA)	Japan
Iyasu, Solomon	Formerly Merck Sharp & Dohme	USA
Jonsson Funk, Michele	University of North Carolina at Chapel Hill	USA
Juhaeri, Juhaeri	Sanofi	USA
Junji, Moriya	Pharmaceuticals and Medical Devices Agency (PMDA)	Japan
Lambert, Laurie	Canadian Agency for Drugs and Technologies in Health (CADTH)	Canada
Li, Jie	Food and Drug Administration (FDA)	USA
de Luise, Cynthia	Pfizer	USA
Machlitt, Andrea	Bayer	USA
Mayer, Miguel‐Angel	Hospital del Mar Barcelona/Universitat Pompeu Fabra Barcelona	Spain
Mera, Robertino	Gilead	USA
Nishioka, Kinue	Pharmaceuticals and Medical Devices Agency (PMDA)	Japan
Nomura, Manami	Pharmaceuticals and Medical Devices Agency (PMDA)	Japan
Nonaka, Takahiro	Osaka Metropolitan University	Japan
Rägo, Lembit	Council for International Organizations of Medical Sciences (CIOMS) Switzerland	Switzerland
Rubino, Heather	Pfizer	USA
Sato, Daisaku	Pharmaceuticals and Medical Devices Agency (PMDA)	Japan
Schiel, Anja	Norwegian Medical Products Agency (NOMA), Scientific Advice Working Party, European Medicines Agency (EMA), and MPG and JSC member HTA‐Coordination Group (HTA‐CG)	Norway
Shaw, David	Maastricht University/University of Basel	Switzerland
Soares, Monica	Brazilian Health Regulatory Agency (ANVISA)	Brazil
Stingl, Julia	University Hospital of Rheinisch‐Westfälische Technische Hochschule (RWTH) Aachen	Germany
Townend, David	University of London	UK
Wakao, Rika	Pharmaceuticals and Medical Devices Agency (PMDA)	Japan
Wang, Shirley	Harvard Medical School	USA
Wicherski, Julia	Federal Institute for Drugs and Medical Devices (BfArM)	Germany
Wormser, David	Novartis Pharma AG	USA
Zint, Kristina	Boehringer Ingelheim International GmbH	Germany

## Conflicts of Interest

The authors declare no conflicts of interest.
